# Novel Biomarkers of Abdominal Aortic Aneurysm Disease: Identifying Gaps and Dispelling Misperceptions

**DOI:** 10.1155/2014/925840

**Published:** 2014-05-20

**Authors:** Demetrios Moris, Eleftherios Mantonakis, Efthymios Avgerinos, Marinos Makris, Chris Bakoyiannis, Emmanuel Pikoulis, Sotirios Georgopoulos

**Affiliations:** ^1^First Department of Surgery, Vascular Unit, “Laiko” General Hospital, National and Kapodistrian University of Athens, Agiou Thoma 17, 11527 Athens, Greece; ^2^Division of Vascular Surgery, University of Pittsburgh, Pittsburgh, PA 15213-2582, USA; ^3^Imperial College, London SW7 2AZ, UK

## Abstract

Abdominal aortic aneurysm (AAA) is a prevalent and potentially life-threatening disease. Early detection by screening programs and subsequent surveillance has been shown to be effective at reducing the risk of mortality due to aneurysm rupture. The aim of this review is to summarize the developments in the literature concerning the latest biomarkers (from 2008 to date) and their potential screening and therapeutic values. Our search included human studies in English and found numerous novel biomarkers under research, which were categorized in 6 groups. Most of these studies are either experimental or hampered by their low numbers of patients. We concluded that currently no specific laboratory markers allow screeing for the disease and monitoring its progression or the results of treatment. Further studies and studies in larger patient groups are required in order to validate biomarkers as cost-effective tools in the AAA disease.

## 1. Introduction

Abdominal aortic aneurysm (AAA) is a relatively common and potentially life-threatening disease, with a prevalence ranging from 4.1% to 11.5% in European populations [1]. The most feared complication of AAA disease is rupture, which often leads to the patient's death. While small aneurysms may rupture too, the risk is greater for bigger aneurysms, so early diagnosis through screening and prompt evaluation of which aneurysms are likely to rupture are key points in the management and research around AAA. No effective conservative treatment exists and, when decided to intervene, surgical correction remains the only effective and “finite” treatment of AAA, with the optimal timing for surgery being the main debatable point. Existing evidence indicates better overall outcome when repair of AAA is performed on elective basis compared to the emergency repair [2–4]. Thus screening programs of AAA have been initiated in developed countries such as UK [5]. Current guidelines recommend elective repair for aneurysms of more than 5.5 cm in diameter, while increased risk groups (women, smokers, and vascular hypertension and chronic airway patients) may benefit by a lower threshold of 5 cm [6].

Advances in the endovascular aneurysm repair (EVAR) technique over the last two decades have led to a major paradigm shift in surgical approach for aortic aneurysms [7, 8]. By providing lower perioperative mortality and morbidity, endovascular techniques are increasingly being preferred in the elective setting [8, 9]. However, late complications of EVAR, such as endoleaks, may result in progression of the underlying aneurysm, even delayed rupture, thus mandating a lifelong surveillance program [10].

Traditional AAA screening, evaluation, and surveillance programs employ the use of imaging techniques such as CT angiogram (CTA), ultrasound sonography (US), or magnetic resonance imaging (MRI). Despite the proven efficacy of these imaging techniques, the cost associated with such programs can incur significant financial burdens to the health care systems [11–14], so alternative methods are continuously being researched.

Biomarkers have attracted much attention in the field of aneurysm research and are defined as measurable molecules (peptides, proteins, and metabolic products) that express a specific biological process in the organism at a given time [15]. In order for a biomarker to be useful, it should be able either to detect the disease itself or to express its progression. So a useful biomarker should detect the presence of a subclinical aneurysm or be a measure of its size and expansion rate, thus predicting the risk of rupture. It is known that this risk increases when the observed expansion rate is greater than the expected [16]. Furthermore, a biomarker could define the optimal surveillance intervals and possibly identify pathogenic pathways which could guide monitoring and treatment [17]. We should not fail to mention that, in order for a biomarker to be employed in modern healthcare system, its use should be cost-effective. Some of the patients selected through this process should be pointed towards more focused screening by specialized imaging techniques. Circulating biomarkers thus present as attractive alternatives for screening and monitoring purposes particularly in healthcare systems which lack the infrastructure to support other primary or secondary screening programs. In this review, we aim to provide a summary of the emerging biomarkers implicated in AAA pathophysiology.

## 2. Materials and Methods

The MEDLINE/PubMed database was searched for publications with the medical subject heading “biomarkers in abdominal aortic aneurysms (AAA).” Two independent reviewers (D.M. and E.M.) performed the literature search, the study selection, and the data extraction. All the references from the identified articles were searched for relevant information. The starting date was January 2008, since, previously, bibliography was covered by the review of Urbonavicius et al. [17] and the end date of the review was April 2014. We focused on human studies that reported the presence of aneurysm, size, expansion rates, and risk of rupture and on new biomarkers and their potential value relevant to the management of AAA. We excluded articles that were not written in English or articles of which full-texts and abstracts were not accessible or comprehensive.

## 3. Results

Numerous studies were found and categorized in the following categories: biomarkers related to the extracellular matrix homeostasis or proteolysis, biomarkers related to cellular signaling pathways, proteins releases by the intraluminal thrombus, biomarkers related to circulating cells and inflammation, metabolomics, and genetic biomarkers. These are described separately in the following section.

### 3.1. Biomarkers Related to Extracellular Matrix Homeostasis or Proteolysis

#### 3.1.1. Cystatin C and Cathepsin

Cystatin C is the endogenous inhibitor of the elastolytic enzymes cathepsins, which are strongly expressed in the aneurysmatic wall. Cystatin C expression is found reduced in AAA disease, leading to lack of inhibition of the elastolytic properties of cathepsins. In a prospective study [18], a negative correlation of serum cystatin C values with AAA size and annual expansion rate was found, but without mentionable potential for predicting cases requiring surgery.

In another large study [19], plasma cathepsin S levels correlated positively with aortic diameters but negatively with lowest ankle-branchial index (ABI). Plasma cathepsin S levels showed no correlation with AAA annual expansion rate, a finding mentioned as inexplicable. After logistic regression analysis, the study also reflected reduced cystatin C in humans with AAA lesions, while no association was found between cystatin C and AAA annual growth rate, likely because of the relatively short AAA observation time.

#### 3.1.2. Circulating Basement-Membrane (BM) Fragments

Types IV and XVIII of collagen are components of the basement membrane. Ramazani et al. [20] have recently shown that AAA patients had significantly increased levels of types IV and XVIII collagen compared with the controls (*P* = 0.005 and *P* < 0.001, resp.). Moreover, AAA patients had significantly increased level of type XVIII collagen (*P* < 0.01) when compared with the peripheral arterial disease (PAD) group [20].

This study was conducted in a small number of patients, indicating that further studies are required to establish the potential role of BM fragments as biomarkers for AAA.

#### 3.1.3. Osteoprotegerin (OPG)

It has been suggested that serum osteoprotegerin (OPG) levels are associated with growth of AAAs while in vitro experiments showed that OPG promotes matrix metalloprotease (MMP) release from monocytes and vascular smooth muscle cells [21, 22]. In a recent study [23], the concentration of aortic wall OPG was positively associated with established markers of AAA severity (cathepsins A, B, and S and the activity of MMP-2 and MMP-9), while it appeared to be associated with lymphocytes and plasma cells. These newer data in humans reinforce the older findings of a role for OPG in AAA pathogenesis.

#### 3.1.4. Tenascin-C (TN-C)

TN-C is a matricellular (extracellular matrix) protein that is synthesized by various cell types including vascular smooth muscle cells (VSMC) in response to inflammatory cytokines and mechanical stress [24]. TN-C is typically synthesized in pathological conditions like wounds, inflammation, and tumorigenesis [25]. No biomarker is available for indicating the pathological status of VSMC and interstitial cells and TN-C may be useful for this purpose, possibly in combination with other inflammatory markers and ECM degradation products. TN-C has the advantage of being deposited locally in the inflammatory lesion (AAA) and it is also released in stable forms into circulation [26].

Further studies are required to elucidate the complete function of TN-C and to evaluate whether serum levels or bioimaging of TN-C is better suited for the assessment of disease activity in human AAA.

### 3.2. Biomarkers Related to Cellular Signaling Pathways

#### 3.2.1. C-Reactive Protein

It is known that C-reactive protein (CRP) is an independent risk factor for arteriosclerosis.

In a cohort study in men with small AAAs [27], CRP levels were found higher in larger AAAs, but no association was found with the aneurysm expansion rate. As a possible explanation, it was suggested that inflammation, as depicted by the levels of inflammatory markers, is more a response to expansion rather than the primary cause.

In another study [28], no correlation was found between levels of circulating CRP and other inflammatory markers and the expansion of small-diameter AAAs, indicating no clinical use of these markers in AAA surveillance.

A recent cohort study [29] investigated the association between plasma CRP levels and AAA diameter and assessed the relationship between the gradient of CRP levels and rates of expansion in asymptomatic AAAs. A statistical association was confirmed between the AAA diameter and high sensitive-CRP (hs-CRP) plasma levels, showing a possible causal association and suggesting hs-CRP plasma level gradient as a marker of disease progression and rate of expansion.

Since these results are conflicting, further, larger studies are required to draw safe conclusions.

#### 3.2.2. Diminished Soluble Tumor Necrosis Factor-Like Weak Inducer of Apoptosis (sTWEAK)

sTWEAK is a type II transmembrane glycoprotein of the TNF superfamily that circulates in plasma [30] and is expressed in SMCs and leukocytes in arterial wall [31]. sTWEAK was found reduced in patients with coronary artery disease [32], carotid atherosclerosis [33], or PAD [34]. Martín-Ventura et al. [35] measured sTWEAK plasma levels in patients with AAA and found that sTWEAK concentrations were decreased in small (≤5 cm, *P* = 0.03) as well as large AAAs (>5 cm, *P* = 0.004) compared with healthy subjects. Moreover, sTWEAK concentrations were negatively associated with AAA size (*P* = 0.008) and AAA expansion rate with 5 years of follow-up (*P* = 0.031) [35].

These results show that sTWEAK is strongly associated with the presence of an aneurysm but fails to differentiate among small and large aneurysms [35].

### 3.3. Proteins Released by Intraluminal Thrombus (ILT)

#### 3.3.1. Peroxiredoxin-1 (PRX-1)

Martinez-Pinna et al. [36] analyzed proteins released by intraluminal thrombus (ILT) with proteomic approach and found that PRX-1 was more released by the luminal layer compared with the abluminal layer of the ILT. Increased PRX-1 serum levels in AAA patients compared with healthy subjects and a positive correlation among PRX-1 and AAA diameter and expansion rate were also found. The combination of PRX-1 and AAA size seems to be significantly predictive of AAA growth [36], establishing PRX-1 as a promising biomarker.

#### 3.3.2. Neutrophil Gelatinase-Associated Lipocalin (NGAL)

NGAL plasma concentrations have been associated with cardiovascular diseases [37]. Polymorphonuclear cells (PMNs) isolated from AAA patients secreted significantly greater amounts of NGAL than PMNs from controls and correlated with retrospective AAA growth [38]. The ILT releases large amounts of NGAL compared to the abluminal thrombus, the aneurysm wall, and the healthy aortic media [38]. Further studies in larger subjects groups are needed to confirm the association between NGAL and AAA presence and growth [38].

It has been suggested that the ILT of AAAs predisposes for enlargement and rupture. The growth of the AAA is dependent on proteolytic degradation of elastin. NGAL can bind to MMP-9 and inhibit its degradation, thereby preserving enzymatic activity [39]. Complexes of NGAL and active MMP-9 were present in thrombus, the interface fluid, and the aneurysm wall. Still, the importance of these observations is unknown and the contribution of the complex NGAL/MMP-9 to the AAA growth should be further evaluated [39].

#### 3.3.3. Insulin-Like Growth Factors and Their Binding Proteins (IGFs and IGFBPs)

Lindholt et al. [40] have evaluated the potential role of IGF-I and IGF-II as biomarkers in 115 patients with AAA, kept under annual surveillance for 10 years. Serum IGF-I correlated positively with AAA size and growth rate (*P* = 0.016 and *P* = 0.004, resp.), findings that persist after adjustment for potential confounders. Serum IGF-I level predicted cases needing later surgery (95% confidence interval (CI):0.52–0.73) [40].

IGFBP-1 was localized in the luminal part of AAA thrombus and IGFBP-1 levels were increased in AAA thrombus conditioned media, compared to media layer and healthy media [41]. It seemed to facilitate the potentiation of ADP-induced platelet aggregation triggered by IGF-1, while its concentrations were significantly higher in large AAA patients compared with control subjects (normal aortic size) (*P* < 0.01). Moreover, IGFBP-1 levels correlated with AAA size (*P* < 0.001), which remained significant after adjusting for risk factors [41].

### 3.4. Biomarkers Related to Circulating Cells and Inflammation

#### 3.4.1. Lymphocytes

Increasing evidence shows that the autoimmune response contributes importantly to the pathogenesis of AAA. More specifically, CD4(+), CD25(+), and FOXP3(+) T regulatory cells (Tregs) were found significantly decreased in AAA patients compared to the control group (*P* < 0.01), indicating impaired immunoregulation [42].

Additionally, the loss of the inhibitory receptor CD31 on peripheral T lymphocytes is found to be associated with the incidence of atherosclerotic complications such as AAA in patients [43]. These findings should be further researched in order to establish potential biomarkers of the natural history of AAA, as well as potential therapeutic targets for the inhibition of the creation and progression of aneurysms.

In another study, increased plasma levels of sCD28 and sCD86 (*P* = 0.0001) and decreased plasma levels of sCTLA-4 (*P* = 0.0018) were found in AAA patients compared with normal individuals. These levels were not related to the patient's age or the size of aneurysm, but there was a significant inverse relationship between the concentrations of sCTLA-4 and sCD80 with matrix metalloproteinase-9 [44].

#### 3.4.2. Monocytes

In a more recent study [45], CD16(+) monocyte subsets were found increased in large abdominal aortic aneurysms and were differentially related to circulating and cell-associated (CD143) biochemical and inflammatory biomarkers. A clinical implication of this study is that, by taking common blood measurements (plasma D-dimer, creatinine, and age to derive eGFR, uric acid, total white blood, and neutrophil counts), one could discriminate AAA patients with different monocyte-dependent inflammatory profiles. This study was hampered by cross-sectional design and the relatively small number of patients, while it was unable to find a clear relation of the size of AAA and the values of circulating monocyte subsets in patients at different stages of expansion of aortic damage.

#### 3.4.3. Progenitor Cells

CD34(+) levels are a known marker of circulating progenitor cells. Van Spyk et al. [46] investigated the role and compared the percentage of CD34(+) cells in AAA disease and peripheral vascular disease (PVD). This small study revealed a lower percentage of CD34(+) cells in AAA patients, compared to PVD patients, concluding that AAA is a less severe vascular disease than PVD. Further study is needed in order to establish CD34(+) cells as a biomarker for risk stratification.

#### 3.4.4. Lymphangiogenesis

A study by Scott et al. [47] attempted to research the relationship between inflammation and neovascularization in AAA tissue. The results showed that the aneurysm wall contained high levels of inflammatory infiltrate, while microvascular densities of blood (*P* < 0.001) and lymphatic (*P* = 0.003) vessels were significantly increased in AAA samples compared with controls. Vascularity as expressed by positive staining for CD31, CD105, and D2-40 correlated positively with inflammation, while increased VEGFR-3 and VEGF-A expression was observed within inflammatory areas of AAAs. These results suggest lymphatic vessel involvement in AAA disease, associated with the extent of inflammation [47].

In a recent study [48], infiltration of lymphatic vessel endothelial hyaluronan receptor- (LYVE-) 1, vascular endothelial growth factor- (VEGF-) C, and matrix metalloproteinase- (MMP-) 9-positive macrophages and podoplanin and Prox-1-positive microvessels were identified by immunohistochemistry in the intima/media in the AAA wall, where hypoxia-inducible factors- (HIF-) 1*α* was expressed. VEGF-C and MMP-9 were not expressed in macrophages infiltrating in the adventitia. With the intraoperative use of indocyanine green lymphography, lymph stasis was revealed in the intima/medial in AAA patients, while fluorescence microscopy confirmed the accumulation of lymph in the intima/media but not in adventitia. These results demonstrate that infiltration of macrophages in intima/media is associated with lymphangiogenesis and angiogenesis in AAA, while there is evidence of inadequate lymph-drainage in the AAA wall.

#### 3.4.5. Catalase

PMNs play a key role in AAA progression. Diminished catalase expression and activity were observed in PMNs from AAA patients compared with controls. Catalase plasma levels were also decreased in large and small AAAs when compared with healthy individuals. This study was also conducted in a very small number of patients [49].

#### 3.4.6. Pathogens

One study [50] showed that* P. gingivalis*, a common pathogen involved in chronic periodontitis, accelerates AAA progression via recruitment and activation of neutrophils, leading to production of NETs which are detectable in the plasma of AAA subjects. These results were confirmed by an experiment in rats. Since repeated subclinical episodes of bacteremia are systematically associated with periodontal diseases,* P. gingivalis* could be therefore a key factor in human AAA progression. Still, more epidemiological and observational studies in humans should be made before organizing therapeutic strategies based on the treatment of periodontal disease to prevent AAA evolution towards rupture.

### 3.5. Metabolomics

Metabolomics stands for sensitive analytical techniques such as metabolic fingerprinting with multivariate analysis. Metabolomics seems to be a good approach to find biomarkers of AAA [51].

Guanidinosuccinic acid (GSA), which is mainly released from the ILT, is highly increased in the plasma of AAA patients when compared to controls. GSA behaves like nitric oxide (NO), due to its vasodilatory actions and its ability to activate the NO generating N-methyl-D-aspartate (NMDA) receptor. After being generated in the ILT, GSA is secreted to blood stream, and the amount of secreted GSA is related to the stage of AAA [52].

Hippuric acid is secreted only by the luminal part of the ILT and was found significantly decreased in the plasma of AAA patients. This observation correlates with the hyperexcretion of hippuric acid in atherosclerotic state [51].

Long-chain acylcarnitines were decreased in the plasma of AAA patients compared to controls. There was a clear decreasing trend with increasing size of aneurysm which may indicate altered fatty acid *β*-oxidation or deficiency of carnitine [51]. Additionally, a significant decrease in sphingosine-1-phosphate (S1P) and sphinganine-1-phosphate in AAA patients was also found. Both molecules are sphingolipids; sphinganine-1-phosphate is a parent of sphingosine and S1P. S1P is a lysophospholipid and significant changes in other lysophospholipids like LysoPEs and LysoPCs are reported in this investigation. A possible explanation for decrease in the amount of LysoPCs/PEs in plasma of AAA patients, with a trend related to the size of aneurysm, is their accumulation in the ILT [51].

#### 3.5.1. Vitamin D Binding Protein (DBP) and Vitamin D

During last decades, there has been a surge of interest in vitamin D and its wide range of health benefits, partially due to the many association studies linking vitamin D status with common human diseases. DBP is the main serum carrier of vitamin D metabolites, with albumin acting as an alternative lower affinity binder [53]. Gamberi et al. [54] showed a negative correlation between DBP and the presence of AAA. Even if its value is not well established yet, DBP is pivotal for vascular remodeling and it may have an important role in the protection of vascular walls.

In a more recent observational study [55], 4233 older men (70–88 years old) participated in a randomized controlled trial of screening for AAA. The study measured their infrarenal aortic diameter by ultrasound and their 25(OH)D plasma levels by immunoassay. An inverse relationship between vitamin D status and the presence of larger AAA was found, along with an inverse dose-response association between 25(OH)D concentrations and the size of AAA, suggesting a role of vitamin D in the severity of aneurysmal arterial disease. Further research is needed to clarify the mechanisms underlying these associations.

#### 3.5.2. Homocysteine

Wong et al. [56] investigated in a cross-sectional study the relationship of homocysteine and the presence and diameter of AAAs in older men (70–88 years old). Plasma total homocysteine (tHcy) was found to be associated with the presence of AAA, while there was also a positive dose-response relationship between tHcy and abdominal aortic diameter. The investigators concluded that further, longitudinal studies and clinical trials of lowering tHcy are required in order to assess if these relationships are causal.

#### 3.5.3. Lipoproteins and Lipoprotein-Related Receptors

Results of a meta-analysis suggest that circulating lipoprotein alpha (Lp(a)) concentrations may be higher in patients with AAA than those in subjects without AAA, thus playing a role in the diagnosis of AAA [57]. Low-density lipoprotein receptor-related protein 1 (LRP1) demonstrated significant association with AAA size (*P* = 0.0042) [58]. In a small pilot study in 12 patients by Chan et al. [59], lipoprotein receptor-related protein 1 (LRP1) expression was found significantly lower in AAA patients than in controls, while no significant correlation was shown between LRP1 expression and the size of AAA (*P* > 0.05). These results suggest that a reduction in LRP1 expression could be associated with aneurysm progression.

Other metabolites used to discriminate the natural history of patients with large aneurysm, small aneurysm, and controls are the metabolites of cholesterol. Decreased plasma levels of those metabolites were observed in patients with large and small aneurysms in comparison to controls [51]. Lower serum HDL cholesterol and higher serum LDL cholesterol may be associated with the presence of AAA [60]. The serum HDL concentrations were lower in patients with AAAs and were independently associated with a reduced risk of having an AAA, in men not receiving current lipid-modifying therapy (95% CI 0.56–0.93 per 0.4-mM increase) and in the total cohort (95% CI 0.63–0.91 per 0.4-mM increase). The concentrations of LDL and triglycerides were not associated with the presence of AAAs [61].

Past studies [62] have revealed decreased low-density lipoprotein receptor-related protein 5 (LRP5) gene expression in peripheral blood cells of AAA patients and an association between decreased expression of LRP5 and increased lipoprotein (a) [Lp(a)] levels in AAA patients. In a recent study [63], LRP5 gene polymorphisms rs4988300 and rs3781590 were found independent genetic markers of AAA, even after adjusting for age, sex, dyslipidemia, hypertension, smoking habit, and chronic obstructive pulmonary disease. AAA patients had significantly higher Lp(a) levels than control subjects (*P* < 0.0001). Further studying of the role of these markers in AAA and of LRP5 gene in Lp(a) catabolism and AAA pathophysiology is necessary.

#### 3.5.4. Phospholipases

Wallinder et al. [64] showed that patients with small AAAs had increased levels of the enzyme glycosylphosphatidylinositol-specific phospholipase D (GPI-PLD) compared with controls without aneurysm by using a proteomic approach, providing some evidence of the value of GPI-PLD as a novel potential plasma biomarker for the detection of AAAs [64]. In the same tone, Golledge et al. found [65] that serum secretory phospholipase A(2) (sPLA(2)) activity is elevated in men with small AAAs but is not associated with AAA progression.

#### 3.5.5. Iron

A recent study by Martinez-Pinna et al. [66] found increased red blood cell- (RBC-) borne iron retention and transferrin, transferrin receptor, and ferritin expression in AAA tissue compared to controls. In contrast, decreased circulating iron, transferrin, mean corpuscular haemoglobin concentration (MCHC), and haemoglobin concentration, along with circulating RBC count, were observed in AAA patients compared to controls, whereas hepcidin concentrations were increased in AAA subjects. Moreover, iron, transferrin, and haemoglobin levels were negatively, and hepcidin positively, correlated with aortic diameter in AAA patients. MCHC negatively correlated with thrombus area in another cohort of AAA patients. Anemia was significantly more prevalent in AAA patients compared to that in patients with atherosclerotic aortoiliac occlusive disease. Finally, the mortality risk among AAA patients with anemia was increased by almost 30 as compared to AAA subjects with normal hemoglobin levels. This study showed that local iron retention and altered iron recycling associated with high hepcidin and low transferrin systemic concentrations could lead to reduced circulating haemoglobin levels in AAA patients and that low haemoglobin levels are independently associated with AAA presence and clinical outcome.

### 3.6. Genetic Biomarkers

#### 3.6.1. Telomere Length

Telomeres are specialized DNA sequences at the ends of linear chromosomes. Telomere attrition is the phenomenon of telomere length “shortening” with each successive cell division, eventually leading to cell senescence and/or apoptosis. These observations can contribute to the estimation of the cellular biological age [67]. In humans, reduced telomere length in circulating leucocytes is associated with premature vascular disease, with the telomere:genomic DNA content being significantly reduced in wall biopsies of AAA compared to normal aortas (*P* < 0.001) [67]. This decreased telomerase endothelial expression implies a protective role of telomerase against AAA formation [68]. Lowest telomere restriction fragment (TRF) length values double the risk of having AAA compared with a mean TRF length in the highest values (*P* = 0.005) [69].

#### 3.6.2. AAA1 Locus on Chromosome 19q13

Several single-nucleotide polymorphisms (SNPs) were nominally associated with AAAs (*P* < 0.05) [70, 71]. The SNPs with most significant *P* values were peptidase D (PEPD) and CD22 [71]. Immunohistochemical staining for CD22 revealed protein expression in lymphocytes present in the aneurysmal aortic wall only and no detectable expression in control aortas [71]. PEPD protein was expressed in fibroblasts and myofibroblasts in the media-adventitia border in both aneurysmal and nonaneurysmal tissue samples [71].

#### 3.6.3. 9p21

AAA is among a number of vascular disorders to be recently associated with a common allelic variant situated on chromosome 9p21 [72, 73]. A significant association between rs10757278-G and the presence of AAA was found (*P* = 0.03), an effect size completely consistent with that originally reported [73]. rs10757278 was not significantly associated with altered AAA growth rate [73]. In a newer study [74], single-nucleotide polymorphisms rs10757278 and rs1333049 of chromosome 9p21-3 region were significantly associated with increased risk of AAA. This study revealed no association between polymorphisms and aortic diameters in AAA patients, while a specific genotype (GG of rs10757278) was suggested to interact with the homocysteine biological pathway to stimulate the presence of AAA. This data emphasized the need to further study the role of these biomarkers.

#### 3.6.4. Chemokine Receptors (CCRs)

Chronic inflammation plays an important role in AAA formation. The chemokine receptors CCR2, CCR5, and CXCR3 are associated with pathways implicated previously in aneurysm pathogenesis. Chemokine receptor-2 (CCR2) is involved in the regulation of the inflammatory response [75]. CCR2 heterozygote V64I polymorphism and allele frequency were more frequently observed in the AAA group (*P* = 0.01, *P* = 0.004) [75], but there was no significant difference with the control group in relation to the Delta32 allele frequency [76].

Table 1 summarizes the published studies on biomarkers in AAA and their clinical value.

## 4. Discussion

In this review article, we aimed to give a description of the emerging biomarkers that can correlate with biological processes associated with the existence (presence/diagnosis) and progression (size/risk of rupture) of abdominal aortic aneurysms (AAAs). Our main goal was to enrich the current knowledge on biomarkers in AAA disease, as it was presented by Urbonavicius et al. [17] in their review at 2008, and analyze the progress made in this scientific field from 2008 to date. As stated earlier, the clinical value of biomarkers is based on their properties to satisfy the goals of early detection (screening), surveillance in terms of early and later progression, and monitoring of the biologic performance of an AAA after surgical treatment. An ideal biomarker should be able to be applied in all of the above. Early detection and close surveillance can greatly benefit patients, since they are referred for elective repair with a lower risk, compared with the emergency setting [2–4]. The mortality of elective AAA repair has been 5% or less, but, once rupture occurs, operative mortality is as high as 48% [77]. Studies have also shown increased cost of open repair of ruptured AAAs (rAAAs) compared to elective open repair; some are even as high as five times more per life saved per year, with the costs from emergency repair of rAAA via EVAR to be even higher [78, 79]. Many developed countries like the USA, UK, and Sweden have established national screening programs for AAA based mostly on ultrasonography with important results in the field of early detection and follow-up [11–14, 80]. Unfortunately, this is not the case in other countries, which cannot afford such high cost of implementing such a wide screening program [80]. More specifically, in a recent study [81], it was shown that the individual cost per invited subject was €60 (US $83.2) and 0.011 additional quality adjusted life years (QALY) were gained per patient in the screened cohort, corresponding to an incremental cost-effectiveness ratio (ICER) of €5673/QALY (US $7870/QALY). These numbers are unlikely to be feasible in a period of world economic crisis. Taking into account the relatively high prevalence of the disease and the high mortality rates in cases of rupture, along with the high ICU and hospital costs [82], even after EVAR, it seems imperative to establish and standardize biomarkers that can be applied in daily practice and be cost-effective.

As presented, numerous biomarkers, related to the AAA disease, are currently being researched. Most of these studies are either experimental or hampered by their low numbers of patients. Still, one can say that biomarkers reflect biological processes associated with a disease, making them more interesting for personalized medicine rather than for a statistical point of view.

sTWEAK is strongly correlated with aneurysm existence or expansion rate but fails to differentiate small to large aneurysms [35]. TN-C contributes in stratifying risk in patients with AAA before intervention or after EVAR [2], but further study is required to elucidate the function of TN-C and to evaluate whether serum levels or bioimaging of TN-C would be suited for the assessment of disease activity in human AAAs.

As far as biomarkers related to ILT are concerned, IGF and PRX-1 seem to be the most promising due to their statistically established correlation with AAA diameter and growth [36, 40].

Genetic factors did not offer statistically significant results, even though genetic biomarkers remain a wide and undiscovered research field especially as far as the application of chromosome 12 loci in AAA, not just in cases of aortic dissection [83].

As far as metabolomics is concerned, they are probably beneficial in terms of early detection of AAA, but it is not clear in literature how they are affected and in turn biased from the metabolic status of AAA patients (many of them are smokers or suffer from hypertension or/and dyslipidemia, which are risk factors for developing AAA). Therefore, identified metabolites could be good targets for the early detection of AAA [51].

However, none of the aforementioned biomarkers can adequately present the combination of all the pathophysiological events that generate and expand an AAA. This means that there is no biomarker simultaneously indicative of ILT presence, inflammation, and proteolysis.

## 5. Conclusions

Biomarkers may help to explain pathological processes of AAA existence and expansion and allow us to find novel therapeutic strategies or to determine the efficiency of current therapies. To date, there are no specific laboratory markers, which allow us to screen for the disease and monitor its progression or the results of treatment. Further studies and studies in larger patient groups are required in order to validate biomarkers as cost-effective tools in the AAA disease. Advances in modern science guide medicine towards minimal or noninvasive techniques for the diagnosis and management of diseases. Surgery and in particular abdominal aortic aneurysms are no exception and less invasive techniques, like EVAR, are already gaining ground, compared to older methods. This technological progress will hopefully make biomarkers a reality for screening, monitoring, and choosing the optimal time of intervention in abdominal aneurysms.

## Figures and Tables

**Table 1:** Summary of the published human studies on novel biomarkers (2008–2014) in AAA and their clinical value.

Category	Biomarker	Author	Year	Study design	Findings	Clinical value
Biomarkers related to cellular signaling pathways	CRP	De Haro et al. [29]	2012	Cohort study	A statistical association was confirmed between the AAA diameter and high sensitive-CRP (hs-CRP) plasma levels. Other studies show controversial results [27, 28]	Aneurysm progression
	sTWEAK	Martín-Ventura et al. [35]	2011	Case control study	sTWEAK plasma levels in patients with AAA compared with healthy subjects. sTWEAK concentrations were negatively associated with AAA size and AAA expansion rate	Aneurysm detection and progression

Biomarkers related to circulating cells and inflammation	Lymphocyte correlated biomarkers	Yin et al. [42]	2010	Prospective study	CD4(+), CD25(+), and FOXP3(+) T cells in AAA patients are found significantly lower than those of the control group	Aneurysm detection
	Monocyte-related biomarkers	Ghigliotti et al. [45]	2013	Case control study	CD16(+) monocyte subsets were found increased in large abdominal aortic aneurysms and were differentially related to circulating and cell-associated (CD143) biochemical and inflammatory biomarkers	Aneurysm size
	Progenitor Cells	Van Spyk et al. [46]	2013	Case control study	A lower percentage of CD34(+) cells in AAA patients, compared to PVD patients, was found	Aneurysm detection
	Lymphangiogenesis	Scott et al. [47]	2013	Case control study	Lymphatic vessel involvement in AAA disease, associated with the extent of inflammation	Aneurysm genesis
		Sano et al. [48]	2014	Case control study	Infiltration of macrophages in intima/media is associated with lymphangiogenesis and angiogenesis in AAA, while there is evidence of inadequate lymph-drainage in the AAA wall	
	Catalase (PMNs)	Ramos-Mozo et al. [49]	2011	Case control study	Diminished catalase expression and activity were observed in PMNs from AAA patients compared with controls	Aneurysm detection
	Pathogens	Delbosc et al. [50]	2011	Experimental study	*P. gingivalis* accelerates AAA progression via recruitment and activation of neutrophils, leading to production of NETs	Aneurysm generis

Proteins released by intraluminal thrombus (ILT)	IGFs	Lindholt et al. [40]	2011	Prospective study	Serum IGF-I correlated positively with AAA size and growth rate	Aneurysm size and progression
		Ramos-Mozo et al. [41]	2012	Case control study	IGFBP-1 concentrations were significantly higher in large AAA patients compared with control subjects	Aneurysm size and thrombus existence
	NGAL	Ramos-Mozo et al. [38]	2012	Case control study	AAA patients secreted significantly greater amounts of NGAL than PMNs from controls and correlated with retrospective AAA growth	Aneurysm detection and progression
	Peroxiredoxin-1	Martinez-Pinna et al. [36]	2011	Prospective study	Increased PRX-1 serum levels in AAA patients compared with healthy subjects and positive correlation among PRX-1 and AAA diameter and expansion rate were also found	Aneurysm detection and progression

Biomarkers related to extracellular matrix homeostasis or proteolysis	Cystatin C	Lindholt et al. [18]	2001	Prospective study	Negative correlation with aneurysm size and expansion, no potential for predicting surgery	Aneurysm size and progression
	Cathepsin S	Lv et al. [19]	2012	Prospective study	Positive correlation with aortic diameters, no correlation with AAA annual expansion rate	Aneurysm size and progression
	Circulating basement-membrane fragments	Ramazani et al. [20]	2011	Case control study	AAA patients had significantly increased levels of types IV and XVIII collagen compared with the controls	Aneurysm detection
	OPG	Koole et al. [23]	2012	Retrospective study	Aortic wall OPG was positively associated with established markers of AAA severity, while it appeared to be associated with lymphocytes and plasma cells	Aneurysm genesis and growth
	Tenascin-C (TN-C)	Greenhalgh et al. [2]	2010	Follow-up study	TN-C is used to stratify risk in patients with AAA before intervention and also after endovascular repair	Aneurysm progression and treatment efficacy

Genomic biomarkers	Telomere length	Wilson et al. [67]	2008	Case control study	Telomere: genomic DNA content being significantly reduced in wall biopsies of AAA compared to normal aortas	Aneurysm detection
	AAA1 locus on chromosome 19q13	Lillvis et al. [71]	2011	GWAS	CD22 revealed protein expression in lymphocytes present in the aneurysmal aortic wall only and not in control aorta. PEPD protein was expressed in fibroblasts and myofibroblasts in the media-adventitia border in both aneurysmal and nonaneurysmal tissue samples	Aneurysm detection
	9p21	Thompson et al. [73]	2009	Cohort study	A significant association between rs10757278-G and the presence of AAA was found (*P* = 0.030). rs10757278 was not significantly associated with altered AAA growth rate	Aneurysm detection
		Wei et al. [74]	2014	Case control study	SNP rs10757278 and rs1333049 of chromosome 9p21-3 region were significantly associated with increased risk of AAA. This study revealed no association between polymorphisms and aortic diameters in AAA patients	Aneurysm genesis
	CCRs	Katrancioglu et al. [75]	2011	Case control study	CCR2 heterozygote V64I polymorphism and allele frequency were more frequently observed in the AAA group	Aneurysm detection

Metabolomics	Lps	Takagi et al. [57]	2009	Meta-analysis	Lp(a) concentrations may be higher in patients with AAA than those in subjects without AAA	Aneurysm detection
		Takagi et al. [60]	2010	Meta-analysis	Lower serum HDL cholesterol and higher serum LDL cholesterol may be associated with AAA presence	Aneurysm detection
		Chan et al. [59]	2013	Case control study	Lipoprotein receptor-related protein 1 (LRP1) expression was found significantly lower in AAA patients than controls, while no significant correlation was shown between LRP1 expression and the size of AAA (*P* > 0.05)	Aneurysm detection
		Giusti et al. [62]	2009	Case control study	Association between decreased expression levels of LRP5 gene in AAA patients	Aneurysm detection
		Galora et al. [63]	2013	Case control study	LRP5 gene polymorphisms rs4988300 and rs3781590 were found independent genetic markers of AAA. AAA patients had significantly higher Lp(a) levels than control subjects (*P* < 0.0001)	Aneurysm detection
	Vitamin D binding protein	Gamberi et al. [54]	2011	Proteomics	Negative correlation between DBP and AAA presence	Aneurysm detection
		Wong et al. [55]	2013	Randomized clinical study	An inverse relationship between vitamin D status and the presence of larger AAA was found, along with an inverse dose-response association between 25(OH)D concentrations and the size of AAA	Aneurysm size
	Homocysteine	Wong et al. [56]	2013	Cross-sectional study	Plasma total homocysteine (tHcy) was found to be associated with the presence of AAA, while there was also a positive dose-response relationship between tHcy and abdominal aortic diameter	Aneurysm detection and size
	Phospholipases	Wallinder et al. [64]	2012	Case control study	Small AAA had increased levels of the enzyme glycosylphosphatidylinositol-specific phospholipase D (GPI-PLD) compared with the controls without aneurysm	Aneurysm size and detection
	Iron	Martinez-Pinna et al. [66]	2014		Local iron retention and altered iron recycling associated with high hepcidin and low transferrin systemic concentrations could lead to reduced circulating haemoglobin levels in AAA patients. Low haemoglobin levels are independently associated with AAA presence and clinical outcome	Aneurysm detection and progression

MMPs: matrix-degrading metalloproteinases; tPA: tissue-like plasminogen activator; TNF-alpha: tumor necrosis factor-alpha; IL-6: interleukin-6; CRP: C reactive protein; hs: highly sensitive; Se: selenium; Lp(a): lipoprotein(a); a1-AT: alpha 1 antitrypsin; NGAL: neutrophil gelatinase-associated lipocalin; CCRs: chemokine receptors.
